# Seminal plasma extracellular vesicles tRF-Val-AAC-010 can serve as a predictive factor of successful microdissection testicular sperm extraction in patients with non-obstructive azoospermia

**DOI:** 10.1186/s12958-022-00978-3

**Published:** 2022-07-22

**Authors:** Xiaoxiao Han, Lin Hao, Zhenduo Shi, Ying Li, Liang Wang, Zhenbei Li, Qiang Zhang, Fangfang Hu, Yijuan Cao, Kun Pang, Zuobin Zhu

**Affiliations:** 1 School of Life Science, Tongji University, Shanghai, China; 2 Department of Urology, Xuzhou Central Hospital, Xuzhou, China; 3 Medical Technology School of Xuzhou Medical University, Xuzhou, 221004 China; 4 Department of Bioinformatics, School of Medical Informatics and Engineering, Xuzhou Medical University, Xuzhou, 221004 China; 5 Center of Reproductive Medicine, Xuzhou Central Hospital, Xuzhou, China; 6 Xuzhou Engineering Research Center of Medical Genetics and Transformation, Key Laboratory of Genetic Foundation and Clinical Application, Department of Genetics, Xuzhou Medical University, Xuzhou, China

**Keywords:** Extracellular vesicles, tsRNA, Non-obstructive azoospermia, Obstructive azoospermia, Biomarker

## Abstract

**Background:**

There is a lack of biomarkers for distinguishing non-obstructive azoospermia (NOA) patients with successful sperm retrieval (Sp+) from those with failed sperm retrieval (Sp-). This study aimed to determine the potential of extracellular vesicles tRNA-derived small RNA (tsRNA) as a novel non-invasive biomarker for successful sperm retrieval by microdissection testicular sperm extraction (mTESE).

**Methods:**

The study included 18 patients with NOA with successful sperm retrieval (Sp+) and 23 patients with NOA with failed sperm retrieval (Sp-), 15 obstructive azoospermia (OA) patients, 5 idiopathic oligospermia (IO) patients, and 12 healthy people. Seminal plasma extracellular vesicles tsRNA levels were used in a two-stage case-control study (screened by tsRNA sequencing on Illumina NextSeq instrument and validated by qRT-PCR). The bioinformatic analysis was performed to determine the role of tsRNA in the pathogenesis of non-obstructive azoospermia.

**Results:**

Two tsRNAs (tRF-Val-AAC-010: AUC = 0.96, specificity = 80%, sensitivity = 95%; tRF-Pro-AGG-003: AUC = 0.96, specificity = 87%, sensitivity = 95%) were found to have high predictive accuracy for distinguishing the origin of azoospermia. In addition, the extracellular vesicles tRF-Val-AAC-010 resulted in high predictive ability (AUC = 0.89, sensitivity = 72%, specificity = 91%, *P* < 0.0001) in predicting the presence of sperm in non-obstructive azoospermia undergoing mTESE. Finally, bioinformatic analysis revealed that tRF-Val-AAC-010 were involved in spermatogenesis.

**Conclusions:**

This study identified that the extracellular vesicles tRF-Val-AAC-010 and tRF-Pro-AGG-003 are biomarkers for the diagnosis of non-obstructive azoospermia, and that tRF-Val-AAC-010 as a potential non-invasive biomarker for predicting the presence of sperm in non-obstructive azoospermia testicular tissue.

**Supplementary Information:**

The online version contains supplementary material available at 10.1186/s12958-022-00978-3.

## Background

Infertility affects about 15% of the world’s population as a reproductive system disorder [1], with the male component accounting for approximately 50% [2]. The prevalence of azoospermia accounts for 10 to 20% of male infertility. Microdissection testicular sperm extraction (mTESE) or testicular sperm aspiration (TESA) is a common surgical procedure that allows azoospermia patients to have their own genetic offspring. Azoospermia can be classified into two groups: obstructive azoospermia (OA) with normal spermatogenesis and the sperm could be obtained by mTESE, and non-obstructive azoospermia (NOA), accounting for 60% of azoospermia but has a 50% sperm retrieval failure rate during the TESA or mTESE procedure [3, 4]. Although testicular volume, serum follicle-stimulating hormone (FSH) levels may be used to differentiate between NOA and OA patients, these markers cannot distinguish between NOA patients with a successful sperm retrieval (NOA-(Sp+)) and those who had a failed sperm retrieval (NOA-(Sp-)) [5].

The pathology of testicular biopsy is the “gold standard” for assessing spermatogenesis status in the testis [6]. However, the invasive procedures not only cause discomfort in patients, but may also cause progressive and irreversible destruction and structural distortion of spermatogenic tubules. The study aimed to identify non-invasive biomarkers that can distinguish the etiology of azoospermia and assess patients with a positive or negative testicular sperm extraction. Most cells could secrete extracellular vesicles with a diameter of 30 to 150 nm and a lipid bilayer membrane structure. In a testicular biopsy, NOA-(Sp-) is significantly different from NOA-(Sp+) [6]. As a result, the extracellular vesicles in seminal plasma produced by NOA-(Sp+) and NOA-(Sp+) are also different. Hence, seminal plasma extracellular vesicles are emerging as a source of potential biomarkers for testicular pathology and physiological conditions.

Several investigations have revealed that the male reproductive tract, including the epididymis, seminal vesicles, and the testis, may produce extracellular vesicles. A recent study demonstrated that extracellular vesicles miRNAs in seminal plasma are markers of azoospermia origin [5]. However, no single miRNA or piRNA in seminal plasma extracellular vesicles demonstrated differential expression in the testicular tissues of NOA-(Sp+) and NOA-(Sp-) [5]. A novel regulatory small non-coding RNA known as tRNA-derived small RNA (tsRNA) has been recently shown to be abundant in human body fluids [7]. tsRNAs are fragments formed by the precise regulation of the processing of tRNA and its precursors. tsRNA can be classified into two categories, tRNA related fragments (tRF) and tRNA halves (tiRNA) based on nucleotide compositions, characteristic sizes, functions, and biogenesis [8]. Moreover, tsRNAs can be categorized as tiRNA, tRF-1, tRF-2, tRF-3, and tRF-5 based on where they map on the mature or precursor tRNA transcript [9, 10].

It has been observed that tsRNAs perform a wide range of physiological roles and are involved in a variety of physiological and pathological processes [11]. The composition and number of tsRNAs are highly dependent on specific cell types and pathological conditions, making them a potential class of biomarkers. tsRNAs have been identified as a cancer survival indicator and a potential diagnostic marker [12]. Recent research has revealed that tsRNAs are conserved in mammalian gametes [13]. Moreover, tsRNAs can be strongly linked to Piwi protein, which is essential for maintaining the normal function of germline stem cells [14]. tsRNAs are enriched in seminal plasma and play a crucial role in sperm maturation and fertilization in mammals [13, 15], implying that tsRNAs might be used as a potential biomarker to predict sperm retrieval rate. The significance of tsRNAs in the azoospermia origin remains unclear. This study aimed to evaluate the predictive potential of tsRNAs in testicular sperm extraction for azoospermic patients.

## Materials and methods

### Subjects of study

The study included 41 NOA patients (including 23 NOA-(Sp-) and 18 NOA-(Sp+)), 15 OA patients, 5 idiopathic oligospermia (IO) patients, and 12 healthy individual with normal fertility (> 60 × 10^6^ sperm/ml). The study was approved by the Research Ethics Commission of Xuzhou Central Hospital (XZXY-LJ-20190110-002) and the written informed consent was obtained from all participants. In this study, 41 NOA patients and 15 OA patients had no sperm in their ejaculate, and the NOA-(Sp+) could obtain a small amount of sperm for fertilization by TESE, while the NOA-(Sp-) had a negative outcome. The IO patients (< 10 × 10^6^ sperm/ml) were also included in the study. All the samples used in the screening and validating stages of this study showed no genetic causes (Y-chromosome microdeletion and chromosomal aberration) and clinical factors (anatomic malformation, varicocele, prostatitis, and hemospermia).

### Extracellular vesicles extraction

The residual cellular debris was removed from the seminal plasma using 0.22 μm filter (Merck-Millipore, Darmstadt, Germany). The exosome isolation kits reagent was then added to the volume of the supernatant following to the instructions of the manufacturer (Umibio, Cat. No: UR52130, China) [13, 15]. After shaking the mixture at 4 °C for two hours, the mixture was centrifuged at 10,000×g for 60 minutes at 4 °C to precipitate extracellular vesicles pellets. Pellets were resuspended with 1 × PBS and filtered with extracellular vesicles purification filter at 3000 x g for 10 minutes at 4 °C. The extracellular vesicles solution was stored at − 80 °C immediately after isolation until the subsequent analyses.

### Nanoparticle tracking analysis (NTA)

The particle size and concentration of extracellular vesicles were determined using nanoparticle tracking analysis (NTA) by ZetaView PMX 110 (Particle Metrix, Meerbusch, Germany) and the accompanying software ZetaView 8.04.02. The extracellular vesicles samples were diluted 5000 times with 1 × PBS buffer (Biological Industries, Israel) to measure the particle size and concentration. NTA measurements were recorded and evaluated at 11 different positions. The ZetaView system was calibrated using polystyrene particles of 110 nm. The temperature was maintained at 23 °C to 37 °C.

### Transmission Electron microscopy (TEM) analysis

Extracellular vesicle suspension (5 μl) was placed on a formvar-Carbon copper grid and 50 μl of 1% glutaraldehyde were added for 5 min. Following that, the copper grid was placed in 100 μl ddH_2_O for 2 min (washed 8 times) and then placed into 50 μl uranium oxalate for 5 min. The copper grid was then treated with 50 μl of methylcellulose solution for 10 min. Finally, the copper grid was dried naturally, and exosomes images were captured using TEM at 80 kV.

### Library preparation and sequencing

Total RNA was extracted from seminal plasma extracellular vesicles. The integrity and quantity of each RNA sample was checked using agarose gel electrophoresis and Nanodrop ND-1000 before sequencing. Based on the previous studies, the library preparation was done in this study [16]. RNA fragments from well-mixed libraries were denatured with 0.1 M NaOH to produce single-stranded RNA molecules, which were then added to the reagent cartridge at 1.8 pM concentration. The tsRNA sequencing (50 cycles) was performed using NextSeq 550 V2 kit (#FC-404-2005, Illumina) following the instructions of the manufacturer.

### Statistical analysis

In this study, FastQC was used to assess sequencing quality, and trimmed 5′, 3′-adaptor reads were aligned allowing only one mismatch to mature tRNA sequences [17]. Reads that did not map were aligned allowing only one mismatch to precursor tRNA sequences using bowtie software [18]. With miRDeep2, the remaining reads were aligned allowing only one mismatch to miRNA reference sequences. The expression profiling of tsRNAs can be calculated using mapped read counts [19]. The differentially expressed tsRNAs were screened based on the counts per million of total aligned reads with R package edgeR [20]. For statistical computing and graphics of the expressed tsRNAs, R-Studio version 1.2.5033 was used to perform principal component analysis (PCA), Pie plots, and Volcano plots.

In this study, potential tsRNAs were screened using deep sequencing technology in a small number of samples, and they had to follow the following criteria: (1) The tsRNAs should show statistically significant differential expressions by non-parametric test (negative binomial distribution test) (*p*-value < 0.05, fold change > 3); (2) The tsRNAs of each sample in one group should be expressed higher or lower than each individual in the other group. qRT-PCR verified the differentially expressed tsRNAs. GraphPad Prism7 was used to calculate the “Area Under ROC Curve (AUC)”, sensitivity, and specificity. The means and standard error are used to represent the continuous variables.

## Results

### Profiling of extracellular vesicles tsRNAs in seminal plasma

**Fig. 1:**
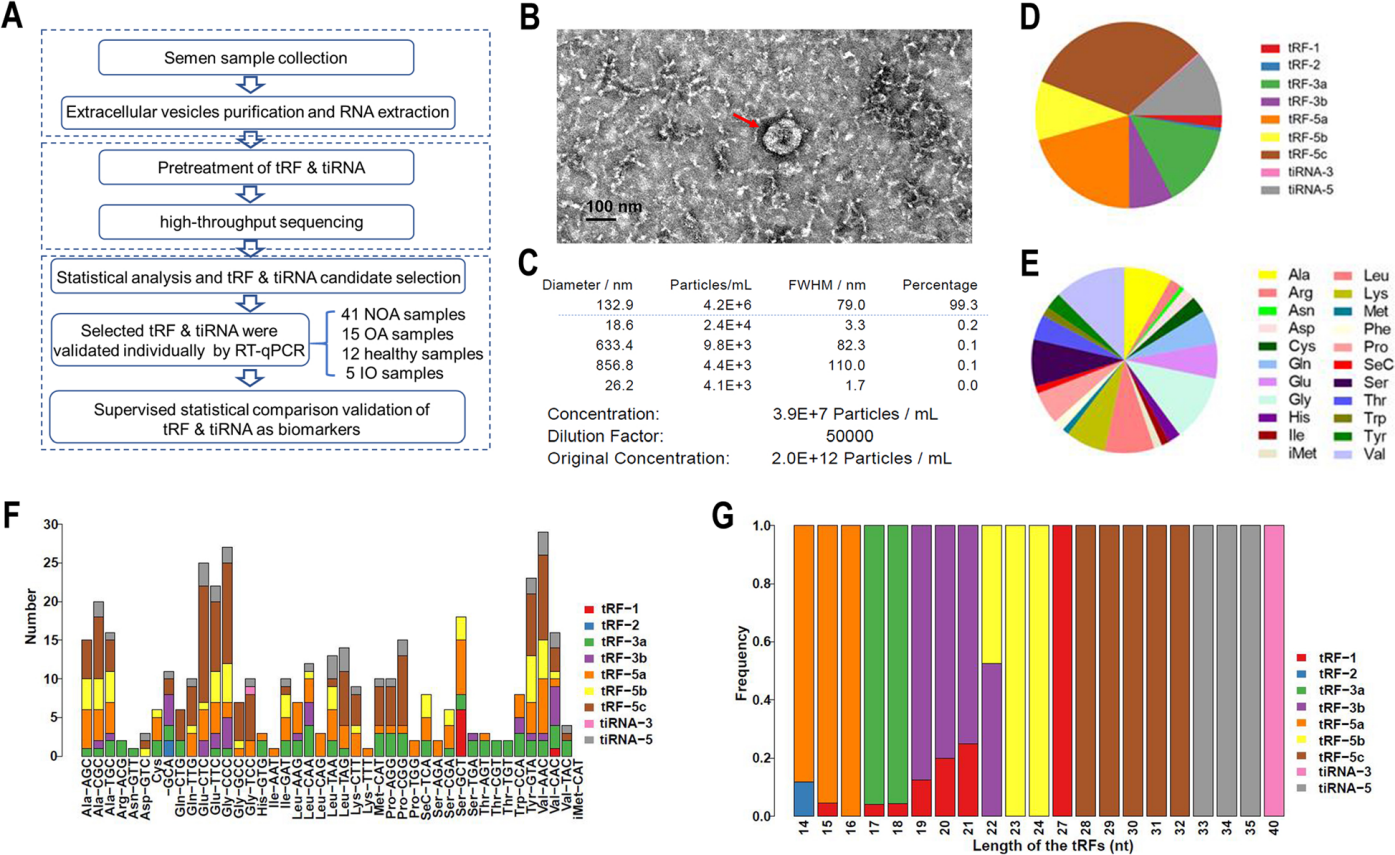
Profiling of extracellular vesicles tsRNAs in seminal plasma. **A** Profiling of extracellular vesicles tsRNAs in seminal plasma. Flow chart of the two stages of tsRNA profiling and validation performed in the study. **B** Transmission electron micrograph (TEM) of extracellular vesicles isolated from seminal plasma (The scale bar is 100 nm). **C** nanoparticle tracking analysis of extracellular vesicles isolated from seminal plasma. **D** Pie char of the distribution of subtype tsRNAs. The values in bracket are represented the number of subtype tsRNAs. The color represents the subtype tsRNAs. **E** Pie char of the distribution of the origin for tsRNAs in seminal plasma

The extracellular vesicles were isolated from seminal plasma and prepared tsRNA library for high throughput sequencing to identify tsRNA as a potential biomarker for NOA. Since the decoration of tsRNAs by RNA modifications interfere with tsRNA-seq library construction [21], total RNA extracted from extracellular vesicles are pretreated to avoid some RNA modifications which interfere with tsRNA-seq library construction and collapsed into FASTA format (https://www.jianguoyun.com/p/DauTV_0QmfLbCRi84YQE). The study detected 400 known tsRNAs and 179 novel tsRNAs. The selected tsRNAs were then validated as biomarkers in a subsequent set of seminal plasma from 61 patients and 12 healthy individuals with normal fertility using RT-qPCR and bioinformatic analysis (Fig. 1A). Supplementary Table 1 displays the baseline clinical disease characteristics. Transmission electron microscopy (TEM) and nanoparticle tracking analysis (NTA) were used to confirm the prepared seminal plasma extracellular vesicles (Fig. 1B and C). NTA showed that the 99.3% of the extracellular vesicles in seminal plasma have a diameter of about 130 nm.

tRF-5 is the most abundant tRNA-derived small RNA in extracellular vesicles (56%), while tRF-2, tRF-1, and tiRNA-3 together account for only 2.9% (Fig. 1D). The composition of nine different tsRNAs were examined to find that the composition for extracellular vesicles tsRNAs in seminal plasma was distributed in 22 tRNAs (Fig. 1E), which differed from the distribution of tsRNAs in plasma such that almost all tRNA-5 s derive from four tRNAs (Gly-, Lys-, Glu-, and Val-tRNA) [12]. The distributions of nine different types of tsRNAs were examined further, and it was revealed that the distributions of each kind of tsRNA displayed significant bias. Surprisingly, a large majority of tRF-5c were enriched in five types of tRNAs (Glu-, Gly-, His-, Lys- and Pro-tRNA); while tRF-1 s were mostly enriched in Ser-TGA tRNA (Supplementary Table 2). The length distributions of tsRNAs were then examined and it was found that tRF-1 were 15–21 nt, tRF-2 were 14 nt, tRF-3a were 17–18 nt, tRF-3b were 19–22 nt, tRF-5a were 14–16 nt, tRF-5b were 22–24 nt, tRF-5c were 28–32 nt, tiRNA-3 were 40 nt and tiRNA-5 were 33–35 nt (Supplementary Table 3). These findings revealed that tsRNAs were not the outcome of random tRNA explanations, but rather may regulate biological function via an undiscovered mechanism.

### Identification of differentially expressed tsRNAs between NOA and OA patients

**Fig. 2:**
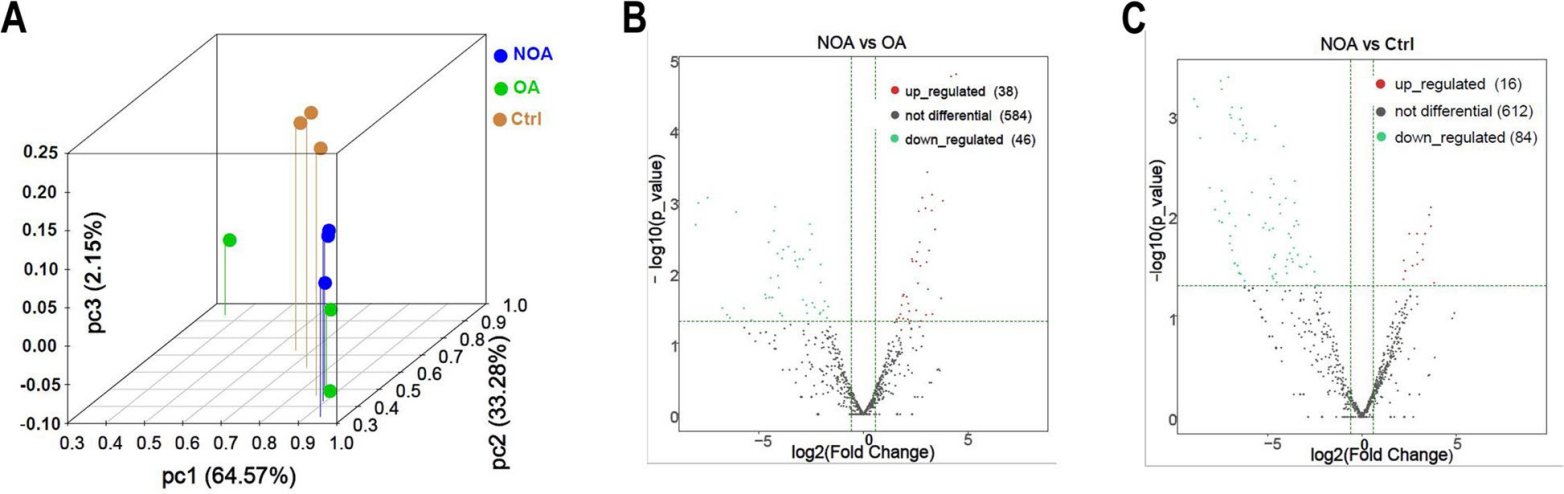
The analysis of expression level in NOA, OA and healthy samples. **A** Primary component analyze. The X, Y and Z axis represents the three main factors which affected the expression level of the sample. The colored point represents the corresponding sample, and the location of it shows the main character of the sample. Space distance represents the similarity of data size. The volcano plot of differentially expressed tsRNAs between NOA and OA patients (**B**) and between NOA patients and healthy people (**C**). The values of X and Y axes in the volcano plot are log2 transformed fold change and -log10 transformed *p*-values between the two groups, respectively. Red/Green circles indicate statistically significant differentially expressed tsRNAs with fold change no less than 1.5 and p-value ⩽ 0.05 (Red: up-regulated; Green: down-regulated). Gray circles indicate non-differentially expressed tsRNAs, with FC and/or q-value are not meeting the cutoff thresholds

To comprehensively profile extracellular vesicles tsRNAs in NOA patients, OA patients, and healthy individuals, the principal component analysis (PCA) method was used which is an unsupervised analysis to reduce the dimensionality of large data sets, and a distinguishable tsRNAs expression profiling among three groups was found (Fig. 2A), indicating that the function of extracellular vesicles tsRNAs maybe associated with spermatogenesis and it can effectively select markers from the tsRNAs profile for differentiating between NOA patients and OA patients. The significantly differentially expressed tsRNAs between NOA patients and OA patients were screened to reveal that 38 tsRNAs were up-regulated and 46 tsRNAs were down-regulated (*P* < 0.05) (Fig. 2B). It was then found that 100 tsRNAs were differentially expressed between NOA patients and healthy people (16 up-regulated, 84 down-regulated) (Fig. 2C) (Supplementary Table 4).

### tRF-Val-AAC-010 and tRF-pro-AGG-003 are non-invasive biomarkers for diagnosis of non-obstructive azoospermia

**Fig. 3:**
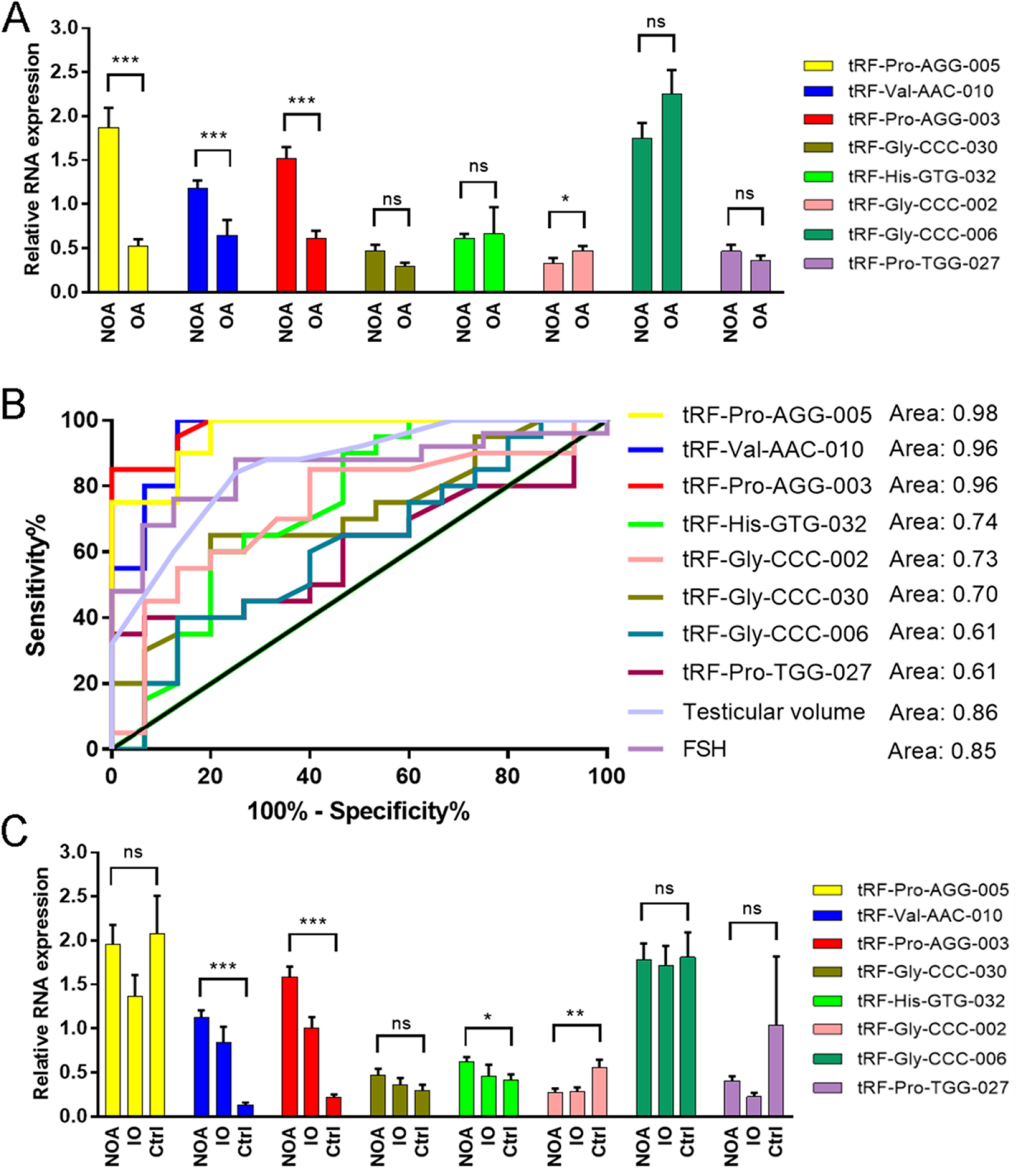
Validation of the eight selected tsRNAs and to assess their predictive efficiency for distinguishing NOA patients with spermatogenic failure from OA patients. **A** Validation of the eight selected tsRNAs by qRT-PCR from NOA and OA. **B** ROC curve of the eight selected tsRNAs for predictive classification of azoospermic patients into NOA and OA subtypes. **C** Validation of the eight selected tsRNAs by qRT-PCR from NOA, IO and healthy samples. Normalized expression levels relative to the U6 gene. Data shown as the means (10–90% percentile). Statistical analysis was performed by using two-tailed unequal variant Student’s t-test (*P-value < 0.05; **P-value < 0.01; ***P-value < 0.001)

To identify the potential biomarker for NOA patients, Eight tsRNAs that showed significant differences in expression when compared with OA patients (difference more than three-fold change; *P* < 0.05, negative binomial distribution test) were selected to identify the potential biomarker for NOA patients. Moreover, these eight miRNAs were further validated individually in subsequent samples (20 NOA samples, 15 OA samples) by RT-qPCR, although only four of them (tRF-Pro-AGG-005, tRF-Val-AAC-010, tRF-Pro-AGG-003 and tRF-Gly-CCC-002) significantly differentially expressed between NOA patients and OA patients (Fig. 3A), the expression values of these four tsRNAs resulted in good predictive accuracy (tRF-Pro-AGG-005 (AUC: 0.98, sensitivity = 95%, specificity = 80%, *P* < 0.0001); tRF-Val-AAC-010 (AUC: 0.96, sensitivity = 95%, specificity = 80%, P < 0.0001); tRF-Pro-AGG-003 (AUC: 0.96, sensitivity = 95%, specificity = 87%, P < 0.0001); tRF-Gly-CCC-002 (AUC: 0.73, sensitivity = 85%, specificity = 60%, *P* = 0.02)) (Fig. 3B). The ROC curve analysis of testicular volume and blood FSH levels, which were the classic predictor for the NOA patients, was also determined, and it was found that tRF-Pro-AGG-005, tRF-Val-AAC-010, and tRF-Pro-AGG-003 as NOA predictors had better predictive accuracy than testicular volume and blood FSH levels (Fig. 3B).

In this study, eight miRNAs were validated in 10 healthy samples and five IO samples, and found that tRF-Val-AAC-010 and tRF-Pro-AGG-003 expression levels were significantly higher when compared to OA samples and healthy samples (Fig. 3C), and tRF-Val-AAC-010 and tRF-Pro-AGG-003 expression levels in IO samples were in the middle of NOA and OA samples. These findings suggest that tRF-Val-AAC-010 and tRF-Pro-AGG-003 are associated with spermatogenesis status.

### Extracellular vesicles tRF-Val-AAC-010 in seminal plasma as a promising biomarker of the presence of sperm in testicular tissue with non-obstructive azoospermia

**Fig. 4:**
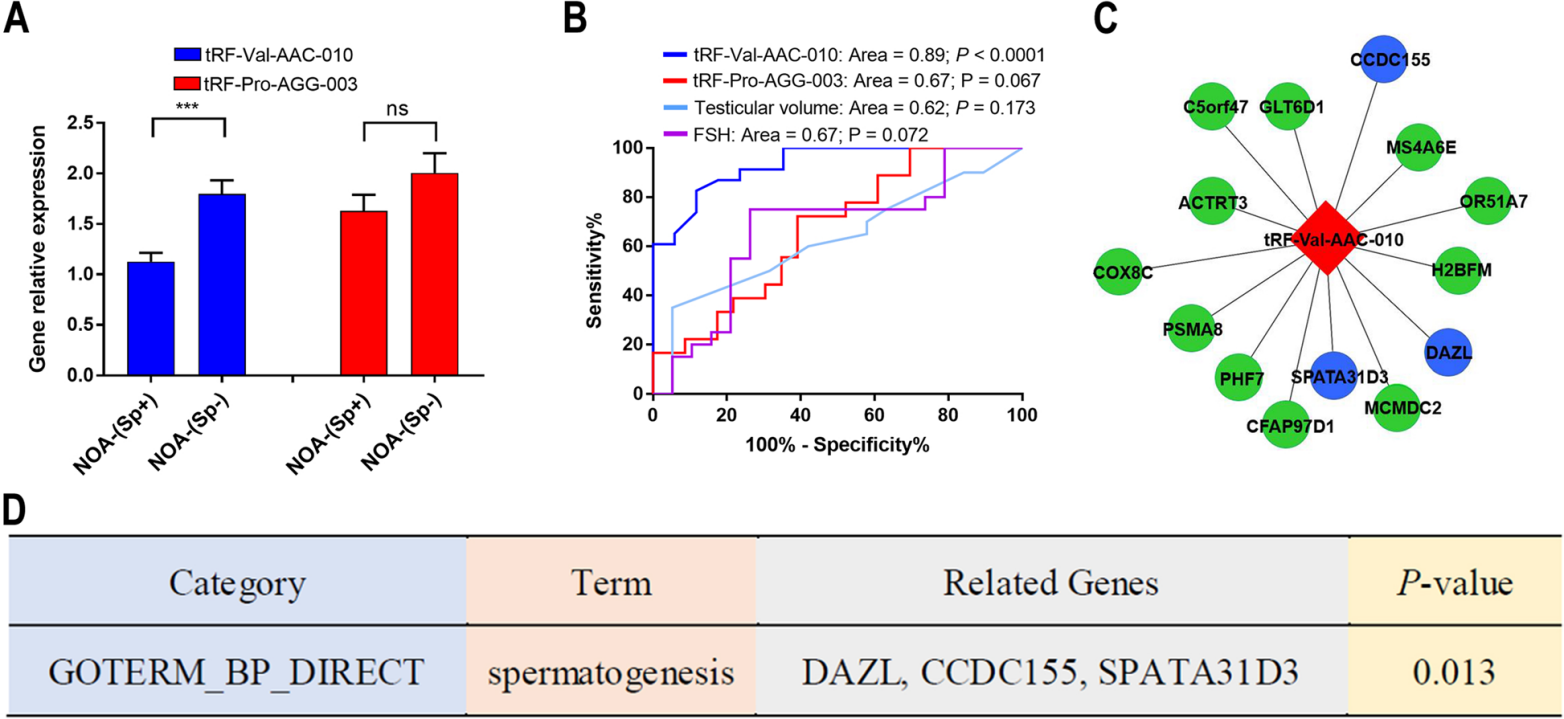
tRF-Val-AAC-010 is biomarker of successful sperm retrieval in testicular tissue with non-obstructive azoospermia. **A** Validation of the expression of tRF-Val-AAC-010 and tRF-Pro-AGG-003 by qRT-PCR from 23 NOA-(Sp-) samples and 18 NOA-(Sp+) samples. Normalized expression levels relative to the U6 gene. **B** ROC curve of tRF-Val-AAC-010, tRF-Pro-AGG-003, testicular volume and FSH level for predictive classification of NOA-(Sp+) patients and NOA-(Sp-) patients. **C** tRF-Val-AAC-010 and its predicted target mRNAs (Nodes in red diamond is tRF-Val-AAC-010; nodes in circle color are predicted target mRNAs for the tRF-Val-AAC-010; nodes of the blue circles are genes that have been reported to be involved in spermatogenesis). **D** The most significant biological process GO terms for the 13 target genes of tRF-Val-AAC-010. Data shown as the means (10–90% percentile). Statistical analysis was performed by using two-tailed unequal variant Student’s t-test (*** *P*-value < 0.001)

The expression of the extracellular vesicles tRF-Val-AAC-010 and tRF-Pro-AGG-003 between 23 NOA-(Sp+) patients and 18 NOA-(Sp-) patients were evaluated to assess whether the biomarkers can predict the chances of successful sperm retrieval in testicular tissue with non-obstructive azoospermia by mTESE. It was found that tRF-Val-AAC-010 expression levels in NOA-(Sp+) samples were increased when compared with NOA-(Sp-) samples (Fig. 4A), resulting in good predictive accuracy (AUC = 0.89, sensitivity = 72%, specificity = 91%, *P* < 0.0001) which also had better predictive accuracy than the testicular volume and blood FSH levels (Fig. 4B). Furthermore, it was confirmed that tRF-Val-AAC-010 and tRF-Pro-AGG-003 can be expressed in testicular tissue of NOA patients (Supplementay Fig. 1). These results suggested that tRF-Val-AAC-010 maybe associated with the quantity of testicular tissue mature germ cells. The study also explored whether the target genes of tRF-Val-AAC-010 are associated with spermatogenesis and found that 13 target genes of tRF-Val-AAC-010 enriched at least four-fold higher in human testis than any other tissue (Fig. 4C) (Supplementary Table 5). The Gene Ontology (GO) analysis of biological process categories showed that the target genes of tRF-Val-AAC-010 are involved in spermatogenesis (Fig. 4D) and mutation in *CCDC155* [22] and *DAZL* [23] genes have also been detected in male infertility patients. These findings further confirm that tRF-Val-AAC-010 are involved in the regulation of spermatogenesis and may be used as potential biomarker to predict the presence of sperm in testicular tissue of NOA patients.

## Discussion

For NOA and OA patients, testicular sperm extraction (TESE) is a significant means of obtaining sperm; however, routine indices such as testicular volume and blood FSH level have limited clinical application for predicting the outcome of TESE for azoospermia [6]. Current studies have shown that seminal plasma contains high concentrations of extracellular vesicles, from different tissue cells in the male reproductive tract [5]. Thus, the extracellular vesicles in seminal plasma can transport various regulatory molecules to the seminal plasma and then reflect the pathophysiological status of the testis.

A recent study showed that no single miRNA and the piRNA in seminal plasma extracellular vesicles could predict the presence of sperm in testicular tissue [5]. The characteristics of tsRNA discovered in seminal plasma extracellular vesicles from NOA and OA patients by deep sequencing are first reported in this study. We aimed to see the potential value of extracellular vesicles tsRNAs for NOA diagnosis by using these tsRNA profiles. Since OA patients have normal spermatogenesis, we believe that if tsRNAs are used as markers for predicting the presence of sperm in testicular tissue, they should first have a significant differential expression between NOA patients and OA patients and healthy people with normal fertility. This criterion is met by tRF-Val-AAC-010 and tRF-Pro-AGG-003. The sperm retrieval success rate is at most 50% during the TESA or mTESE procedure (3, 4). In this study, tRF-Val-AAC-010 performed well in predicting sperm retrieval success rate with specificity 91% and sensitivity 72%. At the same time, tRF-Val-AAC-010 was also differentially expressed in testicular tissue of NOA-(Sp+) patients and NOA-(Sp-) patients, indicating that tRF-Val-AAC-010 in seminal extracellular vesicles could be served as a non-invasive biomarker to predict sperm retrieval rate in testicular tissue with non-obstructive azoospermia undergoing microdissection testicular sperm extraction.

Another requirement for tsRNAs to be a useful clinical indicators for NOA is that they should be involved in NOA development. We predicted that target genes of tRF-Val-AAC-010 since tsRNAs have a role in biological processes mostly through target genes, and found that 13 targets genes were significantly enriched in the testicles (At least four-fold higher mRNA level in the testis compared to any other tissue). Further analysis of the GO terms for the 13 target genes showed that its biological process is significantly enriched in spermatogenesis. Our findings indicate that tRF-Val-AAC-010 may play a role in the biological process of spermatogenesis.

## Conclusion

This study performed a thorough analysis of extracellular vesicles tsRNA expression in detecting NOA-(Sp+) and NOA-(Sp-) patients. Finally, the subsequent functional prediction provides strong evidence for further etiology study of NOA. Although our findings imply that tRF-Val-AAC-010 can be used as a promising biomarker for predicting the presence of sperm in testicular tissue, they should be validated in large samples from different countries or regions before clinical application.

## Supplementary Information

**Additional file 1: Supplementary Fig. 1.** Relative expression of tRF-Val-AAC-010 and tRF-Pro-AGG-003 in exosomes from NOA patients testis.

**Additional file 2: Supplementary Table 1.** The baseline clinical disease characteristics of NOA, OA, IO and control samples.

**Additional file 3: Supplementary Table 2.** The number of subtype tsRNAs against tRNA isodecoders.

**Additional file 4: Supplementary Table 3.** The Frequency of Subtype against Length of the tRF & tiRNA.

**Additional file 5: Supplementary Table 4.** Differentially_Expressed_tRF between between NOA patients and healthy people.

**Additional file 6: Supplementary Table 5.** Target genes of tRF-Pro-AGG-003 and tRF-Val-AAC-010 enriched in the testis.

## Data Availability

The datasets used and analyzed during the current study are available from the corresponding author on reasonable request.

## Abbreviations

tsRNA: tRNA-derived small RNA
mTESE: microdissection testicular sperm extraction
NOA: non-obstructive azoospermia
OA: obstructive azoospermia
IO: idiopathic oligospermia
NOA-(Sp+): NOA patients with a successful sperm retrieval
NOA-(Sp-): NOA had a failed sperm retrieval
tRF: tRNA related fragments
NTA: nanoparticle tracking analysis
