# Supplementary figures and images for: Seminal plasma extracellular vesicles tRF-Val-AAC-010 can serve as a predictive factor of successful microdissection testicular sperm extraction in patients with non-obstructive azoospermia
Source: Reprod Biol Endocrinol. 2022 Jul 22;20:106. doi: 10.1186/s12958-022-00978-3 (PMC9308200; doi:10.1186/s12958-022-00978-3)

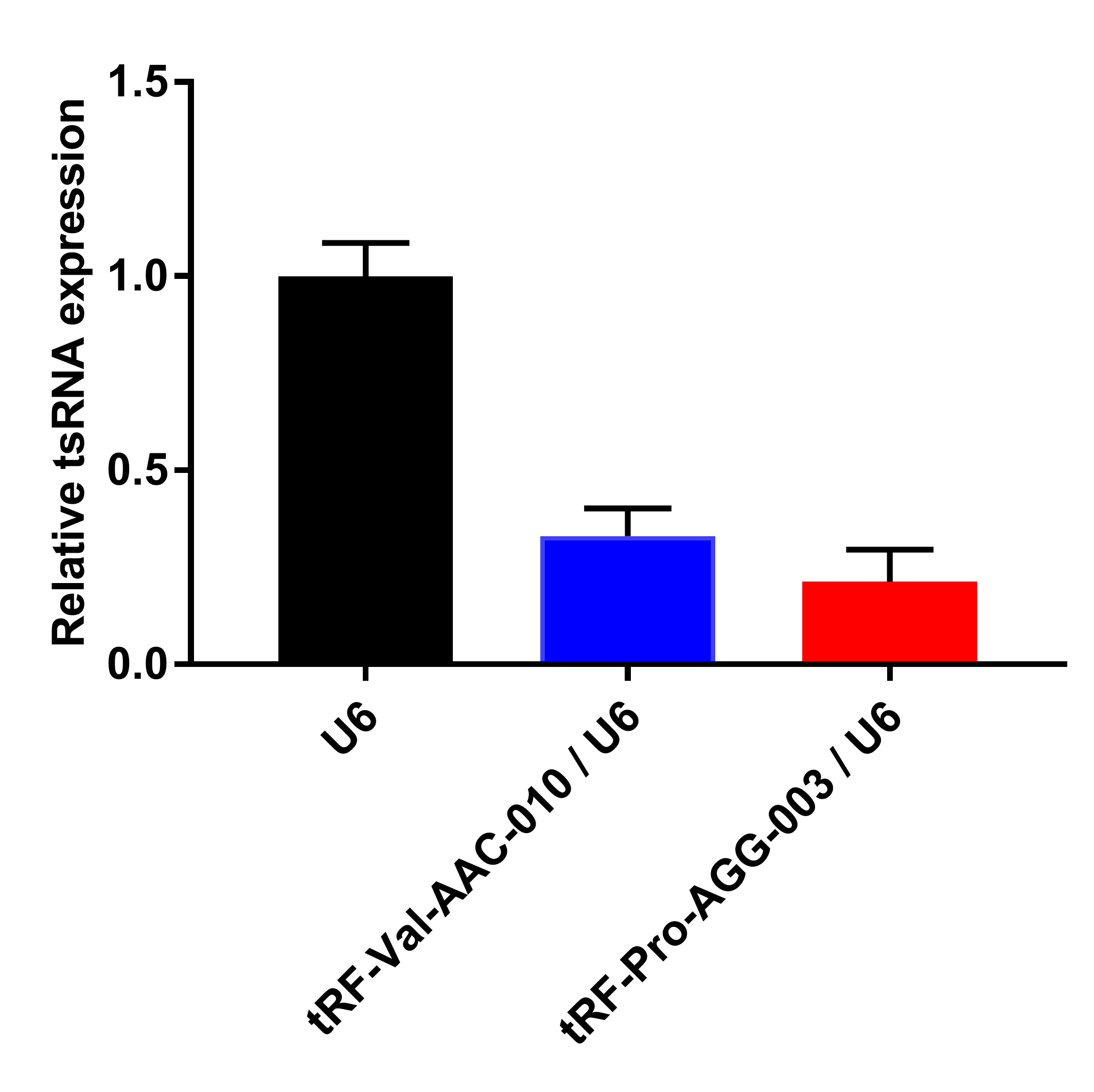

Supplement: Supplementary file 1 — Additional file 1: Supplementary Fig. 1. Relative expression of tRF-Val-AAC-010 and tRF-Pro-AGG-003 in exosomes from NOA patients testis. [file 12958_2022_978_MOESM1_ESM.tif]
